# Laparoscopic Hepatectomy for Hepatic Colorectal Metastases – A Retrospective Comparative Cohort Analysis and Literature Review

**DOI:** 10.1371/journal.pone.0060153

**Published:** 2013-03-21

**Authors:** Jianguo Qiu, Shuting Chen, Prasoon Pankaj, Hong Wu

**Affiliations:** Department of Hepatobiliary Pancreatic Surgery, West China Hospital, Sichuan University, Cheng du, Sichuan Province, China; St. Luc University Hospital, Belgium

## Abstract

**Background:**

Laparoscopic hepatectomy (LH) for management of hepatic colorectal metastases (HCRM) is commonly being performed; however, there are limited reports comparing LH outcomes with those of open hepatectomy (OH) procedure. The aim of the present study was to compare perioperative outcomes between the LH and OH procedures performed at a single medical center.

**Methods:**

From Jan 2008 to May 2012, 30 patients with pathologically confirmed HCRM underwent LH, and 140 patients underwent OH at our hospital. Patients' demographics, perioperative outcomes were retrospectively analyzed.

**Results:**

2 patients (6.7%) in the LH group underwent laparotomies for intraoperative hemorrhage. The LH group had an increased surgical duration (235 min vs. 365 min, (P<0.001), shorter hospital stay (7.5 days vs. 11.5 days, P<0.001), and fewer complications (26.2% vs. 55%, P<0.001) than the OH group. However, in a matched cohort comparison of 30 LH cases and 30 OH cases, no significant variations were observed in the following parameters: surgical duration (235 min vs. 255 min, P = 0.23), positive margin rates (6.7% vs. 0.0%, P = 0.27), or postoperative hematological changes. LH patients had less estimated blood loss (215 ml vs. 385 ml, P<0.001), less morbidity (26.2% vs. 50%, P = 0.02), shorter hospital stay (7.5 days vs. 11.5 days, P<0.001), and lower analgesic requests than with those in the OH group.

**Conclusions:**

LH for metastatic colorectal cancer is a safe and feasible treatment, even in patients who underwent prior laparotomy surgeries and provides significantly less morbidity and shorter hospital stay than OH, without compromising curability or increasing morbidity.

## Introduction

The incidence of colorectal cancer has increased 2–4 folds during the past few decades throughout Asia [1], and 20%–25% of men and women present with hepatic metastasis upon initial diagnoses of colorectal carcinoma [2].

Because of recent technological advances and improved surgical techniques, laparoscopic surgery is routinely performed by surgeons at many centers worldwide [3]–[4]. There is increasing evidence supporting this procedure to significantly improve the immediate outcome of several surgical procedures in various fields of surgery by reducing postoperative pain, hospital stay duration, and allowing for a prompt recovery of daily activities [5]–[10]. However, the number of laparoscopic hepatectomy (LH) procedures performed for hepatic colorectal metastases (HCRM) remains limited and all published reports have been based on limited experience [11]–[16]. Moreover, due to the technical difficulty and insufficient data related to the procedure's oncologic adequacy. Minimal invasiveness of LH weighed against open hepatectomy (OH) technique even less revealed [14]–[16].

Therefore, in the current study, we present our initial experiences with total LH for the management of HCRM and examined the perioperative and oncological outcomes of LH compared to those of conventional OH procedures. In addition, a matched cohort comparison was performed to assess true outcome differences between the LH and OH procedures.

## Patients and Methods

### Patient Population

The protocol conformed to the ethical guidelines of the 1975 Declaration of Helsinki as reflected in a priori approval by the Clinical Trial Ethics Committee of West China Hospital, Sichuan University. A prospective database comprising demographic information, perioperative parameters, and complications of all hepatectomy cases performed at our institute has been maintained since 2003. LH was first performed at our institute in 2008; hence, only patients who underwent LH since that time were included in this study. All cases were required to meet the following eligibility criteria for tumor resection: (1) lesions not associated with major vascular structures; (2) tumor number, ≤5 and the largest tumor ≤5 cm in diameter; (3) absence of other distant/organs metastases via preoperative radiological diagnosis; (4) indocyanine green retention at 15 min (ICG-R15) <15%; and (5) in addition to LH patients, tumor(s) located in the left or right peripheral segments of the liver (Couinaud segments II–VI).

From Jan 2008 to May 2012, 170 HCRM patients, who were accepted by the Division of Hepatobiliary Pancreatic Surgery within our institution, underwent liver resection. Of these patients, 30 patients (17.6%) underwent LH and 140 patients (82.4%) underwent OH.

Demographic, surgical, and perioperative outcomes were retrospectively accumulated. Besides monitoring treatment characteristics such as surgical time and estimated blood loss, other operative parameters, including conversion to an open procedure, hand-port use, and the portal vein clamping method was also documented. Similarly, pathologic details pertaining to tumor size and tumor number were collected.

### Definition

Patients with synchronous colorectal metastases were considered for surgery if their lesions transpired within 6 months after resection of the primary tumor and those who underwent surgery within 3 months from a preliminary diagnosis of liver metastases.

Liver resection cases categorized according to Couinaud's classification as follows: (1) left hepatectomy for resection of segments II–IV; (2) right hemihepatectomy for resection of segments V–VIII; (3) bisegmentectomy for resection of segments II and III; (4) segmentectomy for resection of a single segment; (5) subsegmentectomy for resection of significantly less than a single segment.

In an effort to standardize credit reporting, the Clavien – Dindo Classification of Surgical Complications was applied to the existing research [17]. Grade 1 complications required no surgical or medicinal treatment; Grade 2 complications required medicinal therapy; Grade 3 complications required surgical, endoscopic, or radiological treatment; Grade 4 complications were life-threatening involving the central nervous system, single organ failure, or multiorgan failure necessitating ICU admission; Grade 5 complications resulted in death of the patient. In the current analysis, grades 1 and 2 were considered as minor complications, whereas grades 3–5 were considered as major complications. Postoperative analgesic requests were assessed by the total dosage and administration duration of analgesics.

### Surgical Techniques

Both LH and OH were executed by our surgical team who has extensive experience with these procedures. The comprehensive LH approaches utilized have been detailed by our group previously [16] and elsewhere [12], [13].

First, the patient was placed in the supine position under carbon dioxide pneumoperitoneum, with the abdominal pressure maintained between 10 and 15 mmHg. A 10-mm trocar was positioned 15 mm above the umbilicus for abdominal exploration. The trocar placement sites relied on the location of the lesion and the surgeon's experience. Four to five operative plugins were placed into the upper abdominal region.

Second, the liver lesions were assessed via laparoscopic ultrasonography for the open surgical procedures to verify the expansion of the cancerous growth, number of lesions, their position relative to the main hepatic structures, and to assess additional liver lesions after liver mobilization. The hepatic pedicle was secured with tape in case a Pringle maneuver became necessary. The resection line was marked on the liver surface by electrocautery following an ultrasonographic examination to locate the tumor(s). An endoscopic ultrasonic knife (Ethicon, Boston, MA, USA) was used for hepatic parenchymal transection. The minor vessels and bile ducts were divided using ultrasonographic scissors, clips, or the LigaSure vessel. Larger radicals of the intrahepatic bile ducts and vasculature were trimmed with laparoscopic clips and an Endo-GIA vascular stapler.

Finally, the resected specimen was placed in a plastic bag after it was withdrawn through a suprapubic incision or a new minilaparotomic incision for pathological evaluation. The operative area was irrigated and inspected for bleeding or bile leakage. Abdominal drainage was performed in 14 patients. The surgery was converted to an open resection if there was concern of an inadequate margin resection using the laparoscopic approach, difficult manipulation, or uncontrolled bleeding.

In the OH group, following a left or right subcostal incision and mobilization of the liver to reveal the lesions visually, the abovementioned laparoscopic procedures were implemented for OH using a water-jet dissector and ultrasonic dissector (Cavitron Ultrasonic Surgical Aspirator; Tyco Healthcare, Mansfield, MA, USA). Bipolar electrocoagulation was performed to reduce blood loss. Intraparenchymal management of the primary vessels was attained with clips or nonabsorbable sutures. Portal triad clamping was prepared in all patients; however, only 10 patients required intermittent portal triad clamping (15 min clamping and 5 min release periods) with an average time period of 20±15 min to control intraoperative blood loss. Peritoneal drainage was performed in 16 patients.

### Unmatched and Matched Comparisons

Clinicopathological differences between the LH and OH patients were evaluated using the following two independent strategies: (1) initially, an unmatched comparison of patients who underwent LH was weighed against patients undergoing conventional OH procedures; (2) a matched cohort comparison to evaluate the true outcome differences between the LH and OH procedures. Six clinical characteristics were chosen to determine the patients included in the matched cohort analysis, which included the Child – Pugh classification, the American Society of Anesthesiologists Physical (ASA) Status, preoperative carcinoembryonic antigen (CEA) level, tumor size (cm), tumor location, and tumor number.

### Statistical Analysis

All values were presented as Mean±SD, the Chi-square test or Fisher exact test were used to evaluate the significant differences between two groups. A value of P<0.05 was considered statistical significance. All analyses were performed using SPSS 16.0 statistical software.

## Results

### All Patients

**Figure 1:**
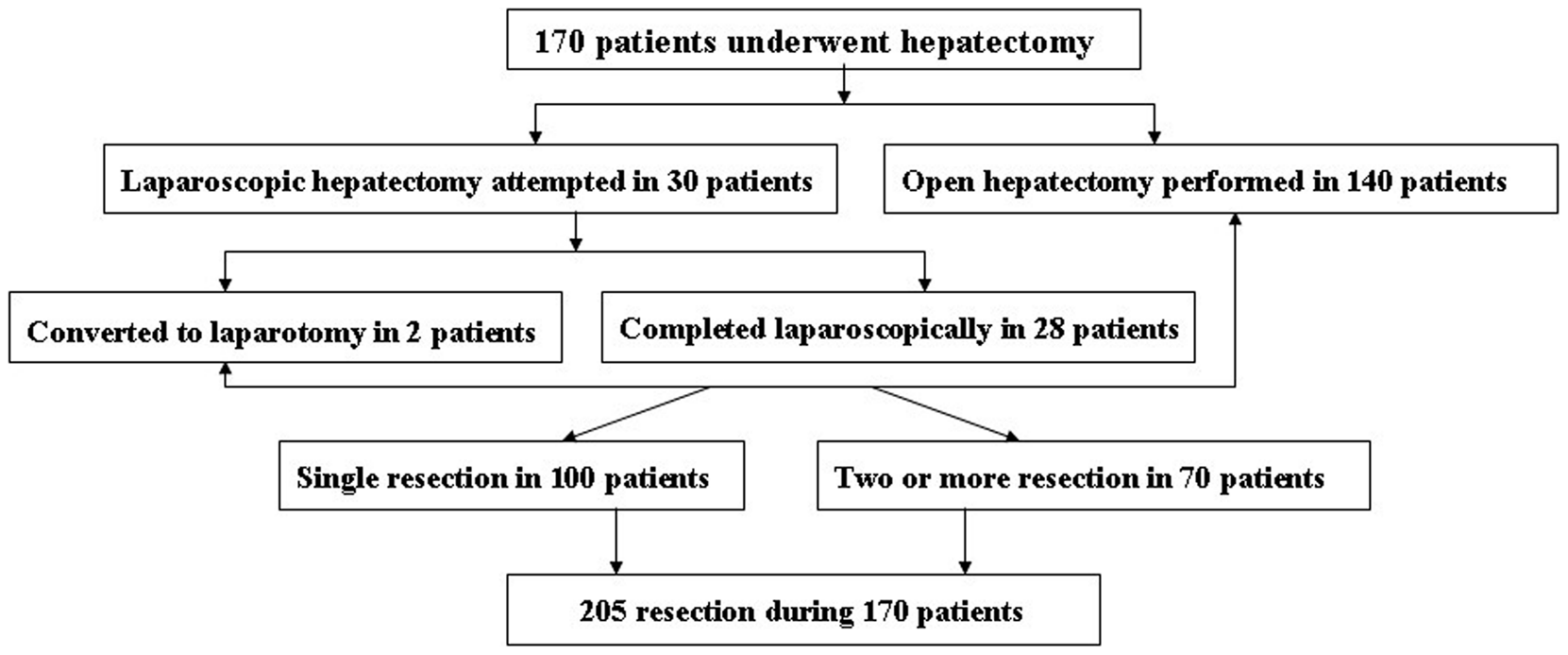
Flow chart of attempted hepatectomy.

Throughout the analysis period, 170 HCRM patients underwent hepatectomies, of which 30 patients (17.6%) underwent LH and 140 patients (82.4%) underwent OH (Figure 1). Characteristics of HCRM patients who underwent hepatectomies are summarized in Table 1. The patient's average age was 53.4±11.7 years, 60.2% were men, and 17 patients had histologically demonstrated cirrhosis. The median tumor size for the group was 3.4 cm (range, 6.8–5.0 cm). The average amount of blood loss in all patients was 358±250 ml and the average duration of hospitalization was 10.4±2.3 days. Of the 170 patients, 65 patients (38.2%) encountered at least 1 postoperative complication, and 15 patients (8.8%) sustained major morbidity. The overall complications rate in this particular series was 39.4% respectively.

**Table 1:** Clinicopathologic characteristics of all included patients.

Clinical characteristics	All (n = 170)	LH (n = 30)	OH (n = 140)	P values (LH vs. OH)
Sex(male/female)	80/90	14/16	66/74	0.96
Age(year)	53.4±11.7	52.5±11.5	54.2±12.5	0.58
Heights(cm)	167.6±6.2	164.5±6.6	168.2±5.7	0.20
Body wight(kg/m^2^)	23±4.5	22.4±5.0	24±7.5	0.14
ASA status(I/II/III)	110/48/12	15/13/2	90/35/15	0.15
Child A/B/C	132/33/5	25/4/1	110/25/5	0.56
Liver cirrhosis(n)	17	2	15	0.23
Primary tumor location				0.56
Rectum	67	11	61	
Colon	89	19	79	
Serum CEA (U/ml)	220±56.8	200±40	220±50	0.25
Tumor location(segments)				0.75
I	3	0	5	
II–III	94	13	72	
IV	20	6	18	
V–VI	45	10	37	
VII–VIII	4	1	8	
Timor size(cm)	3.4±1.8	2.5±2.0	3.7±2.5	0.004
Metastatic tumor number(1/≥2)	100/70	20/10	80/60	<0.001
Tumor presentation				0.55
Synchronous	49	10	39	
Metachonous	121	20	101	
Preoperative chemotherapy	95	13	85	0.08
Postoperative chemotherapy	130	17	110	0.01
Operative time(minutes)	340±80	235±70	365±90	<0.001
Blood loss(ml)	358±250	215±170	390±240	<0.001
Dose of analgesic (mg)	50.2±35.4	30.2±20.8	76.1±44.6	<0.001
Times analgesic given	3.3±0.6	2.0±0.5	5.0±0.8	0.02
Margins(1–10mm/>1cm)	16/154	2/28	14/126	0.57
Specimen weight(g)	310±95	245±45	345±110	<0.001
Surgical margins(mm)	10±6	9±5	12±7	0.006
Hospital stay(d)	10.4±2.3	7.5±1.5	11.5±3.0	<0.001
Overall complication rate	39.4%	26.2%	55%	<0.001

* CEA:Cancer Embryo Antigen.

### Unmatched Patients

Data for unmatched comparison of 30 (17.6%) LH and 140 (82.4%) OH patients are also listed in Table 1. Resected hepatic specimen mass was greater in the OH group, indicating an increased bias for tumor variety than that in the LH group. The median operative time was 235 min (range, 130–330 min) in the LH group and 365 min (range, 110–420 min) in the OH group (p<0.05). Three (10%) LH patients required a mean red blood cell (RBC) transfusion of 0.5±1.5 units, whereas 35 (25%) OH patients required a mean RBC transfusion of 1.2±3.0 units (p<0.05). The estimated mean blood loss was 215±170 ml in the LH group and 390±240 ml in the OH group (p = 0.01). The OH patients experienced significantly longer duration of hospital stays than the LH patients (7.5±1.5 vs.11.5±3.0 days, p<0.001). The complication rate was 26.2% in the LH group and 55% in the OH group (p<0.05) respectively.

### Matched Patients

Data for the a matched comparison of 30 (17.6%) LH and 30 (21.4%) OH patients are listed in Table 2. The resection types were similar between the two groups. Liver metastases presented simultaneously with a primary tumor in 21 patients (35%), and hepatectomy was performed within 12 months following primary tumor resection in 34 patients (56.6%). However, two patients (6.6%) in the LH group underwent open procedure due to intraoperative hemorrhage.

**Table 2:** Characteristics comparison of the 30 patients who underwent LH with matched 30 patients who underwent OH procedure.

Clinical characteristics	LH (n = 30)	OH (n = 30)	P values
Sex(male/female)	14/16	15/15	0.61
ASA status(I/II/III)	15/13/2	16/11/3	0.80
Child A/B/C	15/13/2	15/12/3	0.64
Liver cirrhosis	2	3	0.64
Primary tumor type			0.79
Rectum carcinoma	10	11	
Colon carcinoma	20	19	
Serum CEA (U/ml)	200±40	224±55	0.30
Tumor location(segments)			0.80
II–III	13	14	
IV	6	6	
V–VI	10	8	
VII–VIII	1	2	
Largest tumor size(cm)	2.5±2.0	2.8±1.5	0.51
Metastatic tumor number(1/≥2)	20/10	21/9	0.78
Tumor presentation			0.79
Synchronous	10	11	
Metachonous	20	19	
Preoperative chemotherapy	13	15	0.61
Postoperative chemotherapy	17	18	0.79

* ASA: American Society of Anesthesiology.

The surgical durations were equivalent between the two groups, but blood loss was approximately 2 folds greater in the OH patients. Of the 30 patients in the LH group, 3 patients (10%) had a positive surgical margin respectively. In contrast to the OH group, all LH patients had negative resection margins (P = 0.21). The total dosage and administration times of analgesics in the LH group were significantly lower than in the OH group. Changes in postoperative liver function were not statistically different between the two groups despite a relatively increased serum aminotransferase level in the LH group compared to that in the OH group (Table 3).

**Table 3:** Operative findings and postoperative laboratory test for matched cohort.

Parameters	LH(n = 30)	OH(n = 30)	P values
Left hemihepatectomy	1	2	0.56
Right hemihepatectomy	1	3	0.32
Bisegementectomy	12	12	1.00
Segementectomy	9	7	0.58
Subsegementectomy	7	6	0.75
Operative time(minutes)	235±70	255±80	0.30
Blood loss(ml)	215±170	385±260	<0.001
Hospital stay(d)	7.5±1.5	11.5±3.0	<0.001
Dose of analgesic (mg)	30.2±20.8	70.3±38.5	<0.0001
Times analgesic given	2.0±0.5	4.0±0.8	<0.0001
Time bowel function returned (d)	1.0±0.9	2.4±1.8	<0.0001
Time to first defecation (d)	2.2±0.7	4.0±1.5	<0.0001
Tiem to toleration of soft diet (d)	1.8±1.2	3.2±1.0	<0.001
Specimen weight(g)	245±45	255±50	0.55
Surgical margins(mm)	9±5	10±5	0.44
Blood test parameters			
ALT(mmol/L, post 1 to 3 d)	250±175	285±180	0.42
AST(mmol/L, post 1 to 3 d)	262±140	291±150	0.65
TBIL(mmol/L, post 1 to 3 d)	25.2±15	27±19.5	0.56

ALT: Alanine aminotransferase; AST: aspartate aminotransferase; TBIL: Total bilirubin; post: postoperative.

The time to bowel function returned (mean, 1.8 vs. 3.2 days), passage of feces (mean, 2.2 vs. 4.0 days), and soft diet tolerance (mean, 1.0 vs. 2.4 days) occurred significantly earlier in the LH group. The mean hospital stay was 7.5±1.5 days (range, 6–10 days) in the LH group and 11.5±2.8 days (range, 7–22 days) in the OH group (p<0.0001).

There was no in-hospital mortality in either group; however, 10 complications were observed in 8 patients (26.6%) in the LH group, compared with 20 complications in 15 patients (50%) in the OH group (p = 0.01). No significant difference in major (grade ≥ 3) complications between the groups was noted. More specifically, postoperative wound infection rates (13.3% vs. 3.3%), and ascites (23.3% vs. 6.7%) were significantly increased in the OH patient population. There were no significant differences between the two groups for postoperative bleeding, bile leakage, pneumonia, reoperation rate, or diarrhea.

## Discussion

The present analysis was performed to assess perioperative and oncological outcomes of LH for metastatic colorectal carcinoma. To standardize the assessment of the variations among LH and OH procedures, we first performed an unmatched comparison of patients who underwent LH to those who underwent traditional OH. Second, we performed a matched cohort comparison to assess the true differences of surgical outcomes between the LH and OH groups. Overall, LH was associated with significantly less analgesic prerequisites, a shorter duration of hospital stay, and fewer complications in comparison with OH group. These above advantages were further verified by the matched cohort analysis without comprising postoperative oncological outcomes or increasing mortality.

The liver is the most common site for hematogenous spread of primary colorectal cancers. Several studies have shown that an untreated disease is rapidly fatal, with a median survival period of 5–10 months. Patients with limited liver metastases, when left untreated, had 1-, 3-, and 5-year survival rates of 77%, 14%–23%, and 2%–8%, respectively [18]–[19]. Such patients are now considered candidates for resection. In 1978, JH Foster demonstrated, in a first report, the potential utility of an aggressive surgical approach, and reported a 5-year survival rate of 20% in 168 resection cases for hepatic colorectal metastases [20].

Considering that the first laparoscopic liver wedge resection was documented in 1992 [21], only limited series of laparoscopic hepatic procedures for HCRM patients have been published that included assessments of feasibility, safety, and adequacy. Nguyen et al conducted a review of all reported laparoscopic liver resections consisting of >2,800 cases worldwide [22], which demonstrated that 50% of hepatic resections were performed for metastatic cancers. Of these cases, 35% were of colorectal cancers. The most relevant series [11]–[16], [23]–[39] retrieved from the PubMed database were reviewed by our group (Table 4), which revealed that LH was associated with a statistically significant reduction in hospital stay, postoperative morbidity, and restoration of bowel function. However, there are no documented port-site recurrence cases in the literature, no differences in the margin status, disease-free survival, or overall survival rates in HCRM patients who underwent LH, compared to those who underwent OH. In the largest series reported by Nguyen et al, 109 patients underwent LH for metastatic colorectal cancer at 4 medical centers in the United States and 2 medical centers in France [33]. The 1-, 3-, and 5-year overall survival rates were 88%, 69%, and 50%, respectively, while the disease-free survival rates were 65%, 43%, and 43%, respectively. Another series conducted by Sasaki et al., Bryant et al., Kazaryan et al., and Topal et al. reported similar 5-year survival rates of 64%, 64%, 51%, and 48%, respectively [29], [32], [35], [16].

**Table 4:** Clinicopathologic of patients who underwent laparoscopic liver resections for colorectal liver metastases searched from Pubmed database.

Study&year	Enrolled Patients	Tumor Diameter(cm)	Morbidity	Mortality	Surgical margins(cm)	Overall survival (%)	Disease-free survival (%)
Gigot[^23^]2000	10	ND	ND	0	ND	ND	ND
Mala[^24^]2002	15	2.6(1–6)	13.3%	0	71%≥1cm	ND	ND
O”Rourke[25]2004	22	ND	ND	0	54%≥1cm	2-year: 75%	2-year: 67%
Dulucp[^26^]2005	11	ND	ND	ND	100%≥1cm	ND	ND
Mala[^27^]2005	42	3 (0.8–15)	94% R0	0	ND	ND	ND
Vibert[^11^]2006	41	3 (1–17)	ND	ND	Median:0.5cm	3-year: 87%	3-year: 51%
Alkari[^28^]2008	20	ND	4%	0	R0: 84.2%	89%^a^	79%^a^
Spencer[^12^]2008	13	ND	ND	0	1 (1–4.5)	ND	ND
Sasaki[^29^]2009	39	3.5±3.0	5.7%	0	0.5 (0 – 4)	5 years 64%	ND
Robles[^30^]2008	21	4	ND	0	R0: 100%	3-year: 80%	ND
Buell[^31^]2008	31	3.6	ND	ND	R0: 100%	ND	ND
Bryant[^32^]2008	22	3 (0.8–7)	ND	ND	2±1.7 mm	5-year: 64%	5-year: 47%
Patriti[^13^]2009	7	ND	0	0	R0: 100%	ND	ND
Nguyen[^33^]2009	109	3 (4–15.2)	11.9%	0	94.4%≥1cm	5-year: 50%	5-year: 43%
Sanchez[^34^]2009	6	5 (2–11)	ND	ND	ND	ND	ND
Kazaryan [35]2010	107	5 (2–11)	11.9%	ND	0.3 (0.5–1.2)	5-year: 51%	5-year: 42%
Airazat [36]2010	96	ND	ND	ND	ND	ND	ND
Hilal[^14^]2010	55	ND	15%	0	1.7 (0.1– 6.0)	90%^b^	ND
Lee[^37^]2010	10	3.9±2.0	10%	0	R0: 100%	ND	ND
Tranchart[^38^]2010	2	ND	0	0	R0: 100%	ND	ND
Huh[^15^]2011	20	2.0 (0.9–5.5)	50%	0	R0: 100%	3-year: 58.7%	3-year: 52.8%
Abu Hilal[^39^]2012	80	2.5 (0.5–10.5)	11%	ND	R0: 96%	78%^c^	ND
Topal[^16^]2012	20	4(4–7)	35%	0	R0: 95%	5-year: 48%	5-year: 43%

a : after follow-up of 22 months.

b : after median follow-up of 22 months.

c : after a median (range) follow-up period of 13.5 (5–36) months.

ND: not dimensioned.

A particular disadvantage of LH for HCRM is the technological difficulties. Laparoscopic liver resection requires extensive experience and expertise than open liver resection. We highly recommend the employment of intraoperative ultrasound examinations to correctly stage the disease and decrease the potential high risk of insufficient tumor clearance in laparoscopic procedures. The first LH for a patient with hepatocellular carcinoma was performed at our institution in 2008; our expertise shows 50 laparoscopic hepatectomies techniques is reported with emphasis on the learning curve.

Yet another potential disadvantage of LH for HCRM is the potential difficulty in exposing tumors located in the posterior or superior portion of the right hepatic lobe. Therefore, the LH procedure is not recommended priority due to difficult to exposure and the difficulty in controlling bleeding when adjacent major vascular structures are injured. Due to our initial experiences, we selected only lesions located in the left or right peripheral segments, which were considered most suited for a laparoscopic procedure, especially during the early phase of the learning curve. Some skeptics might argue that straight-forward laparoscopic resections are being offered a laparoscopic approach, and the more difficult cases are being done with open surgical procedures. Nevertheless, this is exactly the take-home point, namely that judgment needs to be carefully exercised in selecting the appropriate cases for laparoscopic resection.

### Conclusions

There are several studies that have described the superiority of laparoscopic surgery for liver resection, however, considering the oncologic features of the metastases, relatively few numbers of papers regarding laparoscopic surgery for colorectal liver metastases had published; long-term results of laparoscopic versus open hepatectomy were even less compared. Despite the small sample, our series certify the feasibility and safety of laparoscopic liver resection for HCRM, and prove that laparoscopy offers a better post-operative course and a shorter hospital stay than the traditional open approach, although our study could be criticized for its patient and treatment selection, as well as the retrospective nature of assessment of outcomes.
